# MicroRNA-506-3p inhibits ovarian cancer metastasis by down-regulating the expression of EZH2

**DOI:** 10.7150/jca.66959

**Published:** 2022-01-04

**Authors:** Yue Sun, Chao Meng, Guoyan Liu

**Affiliations:** 1 Department of Gynecology and Obstetrics, Tianjin Medical University General Hospital, Tianjin, 300052, China.; 2 Tianjin Key Laboratory of Female Reproductive Health and Eugenics, Tianjin, 300052, China.

**Keywords:** miR-506-3p, EZH2, ovarian cancer, metastasis

## Abstract

**Objective:** To investigate the role of miR-506-3p in ovarian cancer (OvCa) metastasis.

**Methods:** We overexpressed miR-506-3p in OvCa cells, and cell migration and invasion capacities were assessed in vitro using Transwell assays and wound healing assay. EZH2 is a target of miR-506-3p. We overexpressed and knocked down EZH2 in SKOV3 cells, and assessed its impact on cell migration and invasion. The orthotopic OvCa mouse models were conducted to confirm the role of miR-506-3p in OvCa metastasis.

**Results:** In this research, we found that miR-506-3p reduced EZH2 expression and obviously suppressed the cell migration and invasion in ovarian cancer (OvCa). Moreover, the knockout of EZH2 mimicked the effect of miR-506-3p on invasion and migration, whereas EZH2 overexpression rescued the inhibitory effect of miR-506-3p. The orthotopic OvCa mouse models and clinical cases also confirmed the negative correlation between miR-506-3p and EZH2 in OvCa

**Conclusions:** MiR-506-3p can suppress cell migration and invasion by targeting EZH2 in OvCa. Our study provides evidence supporting miR-506-3p-based therapy in OvCa.

## Introduction

MicroRNAs (miRNAs) are small, endogenous non-coding RNA molecules that bind to the 3′-untranslated region (3′-UTR) of target genes to regulate 30% of the transcriptome, thereby causing mRNA degradation or inhibiting protein translation 1. Numerous studies have shown that miRNA is a vital regulator of many cellular process including fat metabolism, cell proliferation, differentiation, and apoptosis 2, 3. Extensive investigations have demonstrated that miRNAs play a significant role in tumorigenesis, and the abnormal expression of miRNAs causes them to function as oncogenes or tumor suppressor genes 4. The expression level of several miRNAs such as miR-21, miR-191, miR-214 and miR-486-5p is usually increased in tumors can be regarded as oncogenes 5-7. Whereas, the expression level of some other miRNAs tend to be downregulated in tumors, such as miR-30a, miR-126, and miR-532, which can be regarded as tumor suppressors 8-10. These data highlight the potential benefits of target miRNAs in cancer treatment.

Ovarian cancer (OvCa) remains the most lethal gynecological malignant tumor. It is estimated that there will be 21,410 new cases and 13,770 deaths in the United States by 2021^11^. Many patients haved advanced stage disease at the time of diagnosis, leading to the high death rate 11. Approximately 70% of patient deaths are from advanced high grade serous OvCa (HGS-OvCa) 12. The 5-year survival rate of patients with advanced-stage OvCa after initial diagnosis is only about 30% 13. Unlike cancers in other organs, OvCa could not only invade adjacent organs directly, but also spread to the entire abdominal cavity through implant 14. Due to the diffuse nature of OvCa, surgery alone can rarely completely remove the tumor. It is necessary to combine platinum-based chemotherapy after surgery. Although 70% of OvCa patients initially respond to treatment, most eventually die from disease recurrence 15. In order to improve the prognosis and develop better therapeutic strategies for OvCa, in-depth research into the molecular mechanism of OvCa metastasis is critical.

Studies have shown that the miRNA cluster miR-506 located on chromosome Xq27.3 can regulate proliferation, differentiation and migration of cells 16. Generally, mature sequences of miRNA are divided into 5p and 3p according the source, and miR-506-3p occupies a dominant position in miR-506. MiR-506-3p dysregulation has been found in many different cancer types. MiR-506-3p is an oncogene of lung cancer and melanoma 17, 18. In contrast, miR-506-3p exerts its anti-cancer function in clear cell renal cell carcinoma, bladder cancer, breast cancer, cervical cancer and OvCa 19-23. MiR-506-3p can inhibit the CDK4/6-FOXM1 axis in OvCa, thereby suppressing cell proliferation and inducing senescence 23. MiR-506-3p can also regulate E-cadherin and vimentin/N-cadherin to inhibit epithelial-mesenchymal transition (EMT) and metastasis 24. However, the possibility that other targets of miR-506-3p mediate OvCa metastasis has not been completely ruled out. Therefore, it is important to further study the mechanism of miR-506-3p in OvCa metastasis.

In this study, we aimed at investigating the role of miR-506-3p in OvCa metastasis. We found that overexpression of miR-506-3p in OvCa cells can effectively suppress cell migration and invasion in vitro and tumor metastasis in nude mice. Furthermore, the enhancer of zeste homolog 2 (EZH2) has been identified as a functional target of miR-506-3p, which is closely related to the potential for tumor metastasis 25-27. Knockout of EZH2 was similar to the effects of miR-506-3p in OvCa, and overexpression of EZH2 could rescue the migration and invasion capabilities that were inhibited by miR-506-3p. The results indicated that miR-506-3p might act as a tumor suppressor in OvCa metastasis by downregulating EZH2, suggesting that it could be used as a potential therapeutic target for OvCa treatment.

## Materials and Methods

### Tumor tissues

The study included 100 HGS-OvCa patients diagnosed by pathological reports in the Department of Gynecology, Tianjin Medical University General Hospital (Table S1*)*. All patients underwent hysterectomy, bilateral adnexectomy, omentectomy, and lymphadenectomy in our center. They received platinum-based chemotherapy after operation. Patients receiving neoadjuvant chemotherapy or with other malignant tumors were excluded. 100 ovarian cancer tissue specimens were taken from surgically resected specimens and made into paraffin sections. The clinicopathological characteristics of 100 patients are shown in Table S1. The study was approved by the Ethics Committee of Tianjin Medical University General Hospital. All patients signed informed consents.

### Cell culture

Human OvCa cell lines (HeyA8, SKOV3) were obtained from the American Type Culture Collection (ATCC, USA). HeyA8 and SKOV3 cells were maintained in RPMI 1640 medium (Gibco, USA), supplemented with 10% fetal bovine serum (Gibco, USA) and cultured at 37℃ and 5% CO2.

### MiRNA Transfection

The miR-506-3p mimic and miR-ctrl were purchased from Guangzhou RiboBio Co., Ltd (RiboBio, China). According to the manufacturer's instructions, the miR-506-3p mimic or miR-ctrl was transfected into the OvCa cells at a final concentration of 20 nM with LipofectamineRNAiMAX (Invitrogen, USA).

### Migration assay

The Transwell insert containing an 8-µm pore filter was purchased from Corning (Corning, USA). The OvCa cells treated with miR-506-3p mimic or miR-ctrl were suspended in 200 μl serum-free RPMI 1640 medium (1×10^5^ cells/well) and inoculated in the upper chamber of a Transwell insert. The medium in the lower chamber had 10% FBS as a chemoattractant. The Transwell insert was placed in an incubator at 37°C and 5% CO_2_ for 24 hours. The cells on the lower side of the membrane were removed and fixed with 4% paraformaldehyde at room temperature (RT). Then, they were stained with 0.1% crystal violet. The number of migrated cells in 5 random fields was counted under the microscope.

### Invasion assay

We pre-coated the upper chamber with 50 μl Matrigel (BD Biosciences, USA) at a dilution of 1:8 in the invasion assay. The subsequent procedures were the same as the migration assay. After 48 hours of incubation, we fixed, stained and counted the invaded cells as described above.

### Wound healing assay

OvCa cells were cultured in a six-well plate at a concentration of 2×10^5^ cells/well. When the cells reach 60%-80% confluence, straight scratch wounds were made with a p200 pipette tip. The wound closure speed was observed and photographed at 18 hours.

### Western blot analysis

Protein samples were extracted according to manufacturer's instruction and quantified with BCA assay. The polyacrylamide gel was prepared that 10% resolving gel was made with stacking gel (5%) on the top and a gel comb (10 wells). Load samples containing equal amounts of protein (30 μg/lane protein from cell lysate) on the gel and electrophoresis was performed to transfer the protein to PVDF membrane for antibody detection. Seal the membrane in 5% skimmed milk dissolved in 1×Tris-buffered saline solution containing 0.05% Tween-20 (TBST) for 1 hour at 25°C. The primary β-actin antibody (rabbit) and EZH2 antibody (rabbit) were diluted 1/1000 in 1×TBST+3% bovine serum albumin and incubated overnight at 4°C. Then wash the membrane 5-10 min three times on a shaker at RT. Dilute the secondary antibodies (1:1000) in 1×TBST and incubate the membrane for 2 hours at RT. ECL system was used for HRP-conjugated secondary antibody to visualize the proteins.

### Gene silencing

The SKOV3 cells were seeded in a 6-well plate at a density of 2×10^5^ /well. When the cell confluency was 60%-80% after about 12-24 hours, the complete medium was aspirated and 1.5 mL of siRNA Transfection Medium was added to each well. Then, the cells were cultured in the incubator and waited for transfection. The following solutions were prepared: Solution 1: add 5 ul LipofectamineRNAiMAX Transfection Reagent (Invitrogen, USA) to 245 ul medium for each transfection; Solution 2: add 5 ul EZH2 siRNA duplex (Genepharma, China) to 245 ul medium for each transfection. Use a pipette to mix the above Solution 1 and 2 gently, and incubate the mixture at RT for 20 minutes. Add the mixture to the well and shake the culture plate back and forth to mix it with the culture medium. The culture plates were incubated in a CO2 incubator at 37℃ for 48 hours. The medium was discarded and replaced with fresh medium for cell migration and invasion assays and western blot analysis.

### Immunohistochemical (IHC) staining

We performed IHC staining on tumor tissues from mouse orthotopic model as well as from 100 patients with HGS-OvCa, both of which have been reported in our previous research 28. EZH2 positivity was defined as immunoreactive cells in the nucleus. Use a 0-12 scoring system to quantify positive cells, multiply by the signal intensity (0, no signal; 1, weak signal; 2, medium signal; and 3, strong signal), and classify positive cells by percentage (0, <5%; 1, 5%-25%; 2, 25%-50%; 3, 50%-75%; and 4, >75%). Our previous research has performed miRNA in situ hybridization, and defined the low and high expression of miR-506-3p as scores of less than 6 and greater than or equal to 6, respectively 28.

### Statistical analysis

The results were expressed as means ± SDs at least 3 independent experiments. Differences between groups were evaluated by SPSS 21.0 with Student's t-test. A *p* value<0.05 was considered statistically different (*p<0.05, **p<0.01, ***p<0.001).

## Results

### MiR-506-3p inhibits the migration and invasion of OvCa cells

**Figure 1:**
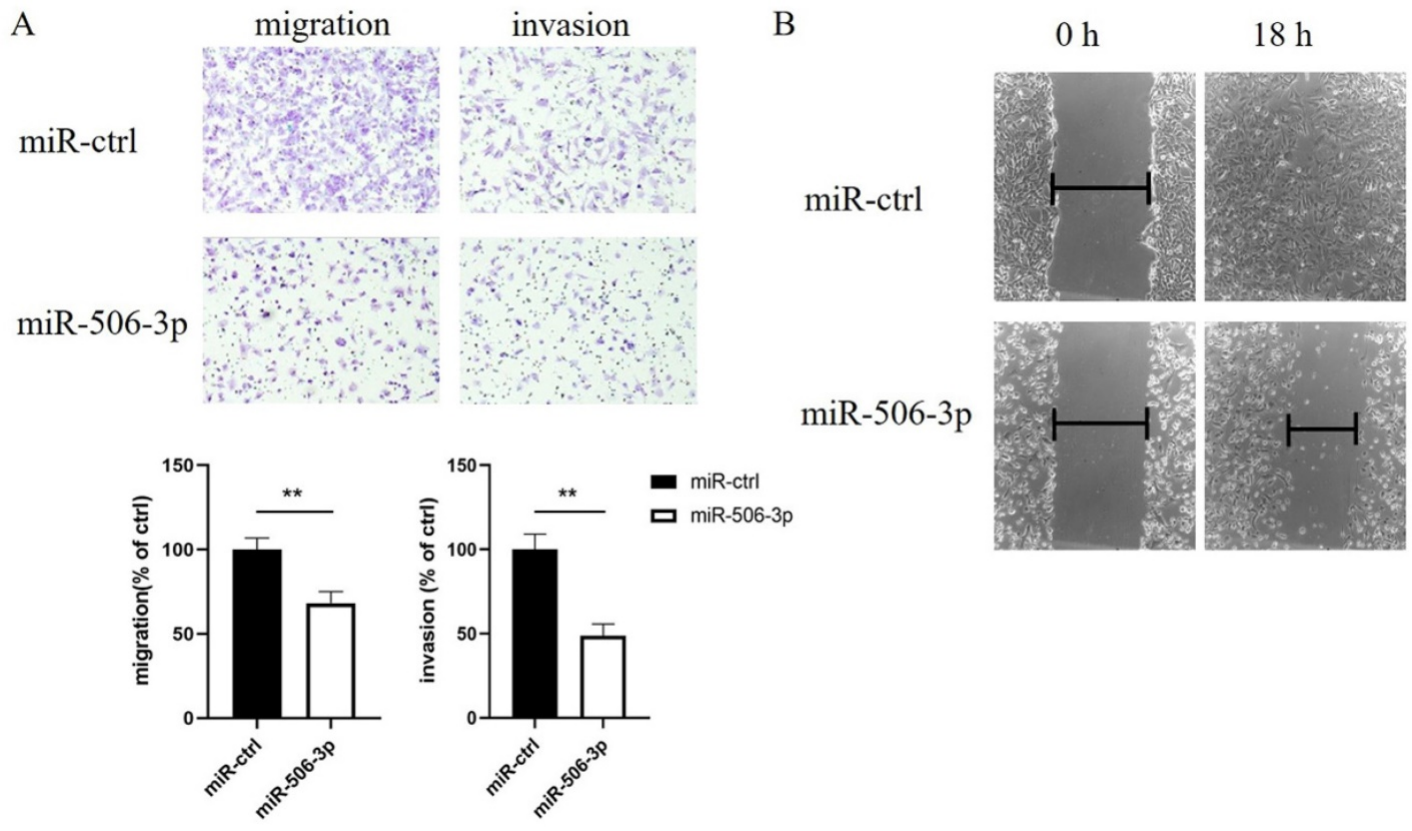
** MiR-506-3p suppresses the migration and invasion of OvCa cells.** SKOV3 cells were transfected with miR-506-3p mimics or miR-ctrl for 48 hours. Transwell assays (A) and wound healing assay (B) were used to assess cell migration and invasion capacities in vitro. (A) Representative images of migrated and invaded SKOV3 cells under the microscope (magnification, x200). The migration and invasion ability of SKOV3 cells after miR-506-3p transfection is lower than that of miR-ctrl transfected cells (**p<0.01). (B) The ability of wound healing was observed under a microscope after 0, 18 hours. The data was analyzed as width ratio (18 hours width/0 hour width). The wound closure rate of SKOV3 cells transfected with miR-506-3p mimics was significantly lower than that of cells transfected with miR-ctrl (**p<0.01).

To explore the effect of miR-506-3p on cell migration and invasion, SKOV3 cells were transiently transfected with miR-506-3p mimic or miR-ctrl. Then, cell migration and invasion capacities were assessed in vitro using Transwell assays. The data indicated that SKOV3 cells treated with miR-506-3p mimic showed lower Transwell migration ability, compared to the cells treated with miR-ctrl (*p*<0.01, Figure 1A). In the invasion assay, miR-506-3p overexpression reduced the invasion ability of SKOV3 cells (*p*<0.01, Figure 1A). These results indicate that miR-506-3p may inhibit cell migration and invasion in OvCa.

### MiR-506-3p inhibits the wound healing of OvCa cells

We subsequently performed a wound healing assay to further confirm that miR-506-3p influenced cell migration. The data revealed that compared to the cells transfected with miR-ctrl, the migratory potential of the SKOV3 cells transfected with miR-506-3p was markedly reduced (Figure 1B).

### EZH2 mediates the effects of miR-506-3p on OvCa cell migration and invasion

**Figure 2:**
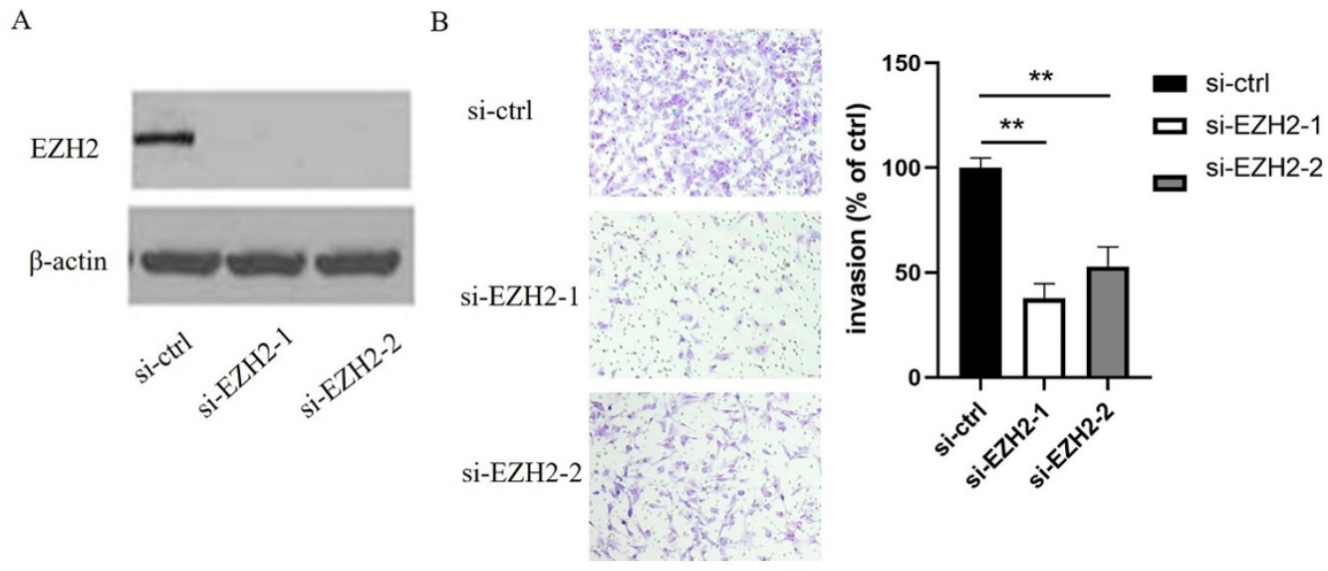
** EZH2 inhibits the migration and invasion of OvCa cells.** (A) The expression of EZH2 after si-EZH2 transfection. HeyA8 cells were transfected with 50nM si-ctrl or si-EZH2, the siRNA efficacy was evaluated by western blot analysis. (B) Transwell assay was used to assess cell invasion. Representative images of invaded HeyA8 cells under the microscope (magnification, x200). HeyA8 cells transfected with si-EZH2 showed less invasive ability than si-ctrl-transfected cells (**p<0.01).

**Figure 3:**
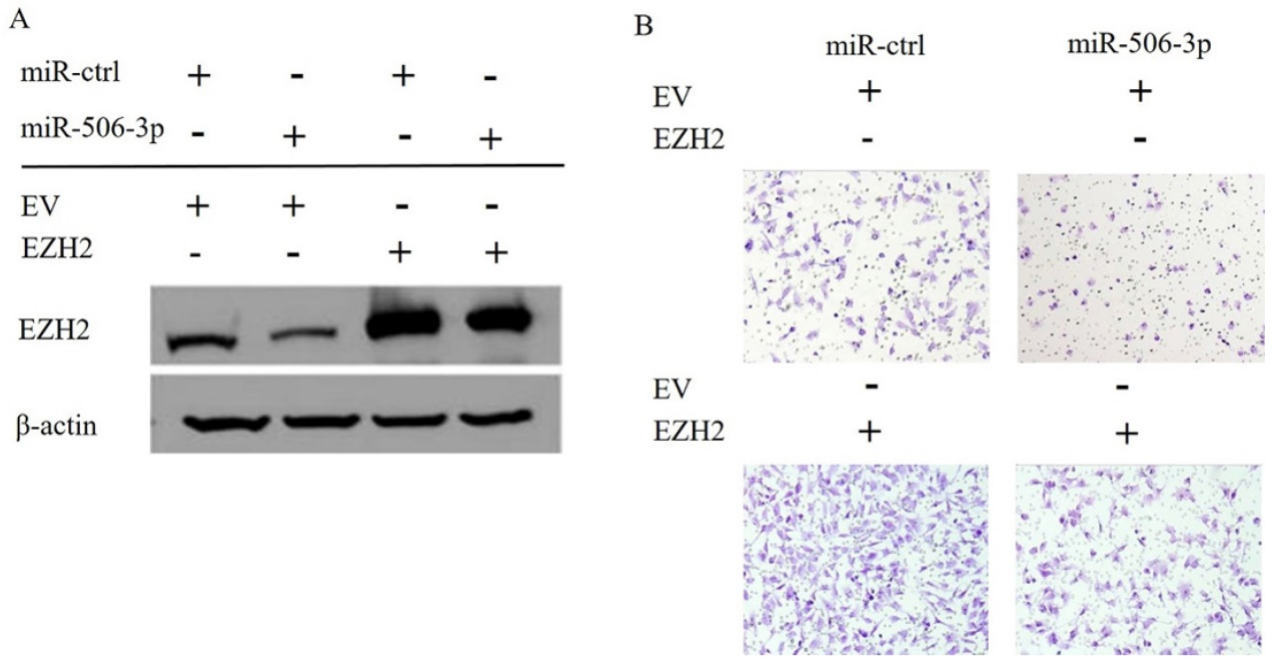
** EZH2 and miR-506-3p induced decrease in migration and invasion of OvCa cells.** SKOV3 cells were co-transfected with empty vector (EV) or EZH2 without the 3'-UTR together with 20 nM miR-ctrl or miR-506-3p. After 24 hours, the cells were harvested for western blot analysis (A) or reseeded for Transwell assay (B).

In order to elucidate the mechanisms by which miR-506-3p regulates cell migration and invasion in OvCa cells, TargetScan software was used to search for the putative protein-coding gene target of miR-506-3p. Our previous studies have shown that EZH2 is a target of miR-506-3p^29^. MiR-506-3p directly targets the 3'-UTR of EZH2, and inhibits the expression of EZH2 gene. EZH2 is a key transcription factor in tumorigenesis and tumor development. Moreover, the upregulation of EZH2 may promote cancer invasion and metastasis 26, 27. Our research also proved that EZH2 is involved in miR-506-3p-mediated cell migration and invasion in OvCa cells. First, we knocked out EZH2 by transfecting siRNA into OvCa cells, and western blot analysis indicated that the EZH2 protein level in OvCa cells was reduced significantly (Figure 2A). Then, as determined in the Transwell assay, knockdown of EZH2 suppressed OvCa cell invasion (*p*<0.01, Figure 2B). Subsequently, we evaluated whether forced EZH2 expression could rescue the inhibitory effect of miR-506-3p. The effect of miR-506-3p on cell migration and invasion could be completely restored by overexpressing EZH2 (Figure 3B). The findings together show that miR-506-3p down-regulates EZH2, thereby inhibiting OvCa cell migration and invasion.

### MiR-506-3p suppresses OvCa tumor metastasis in nude mice

**Figure 4:**
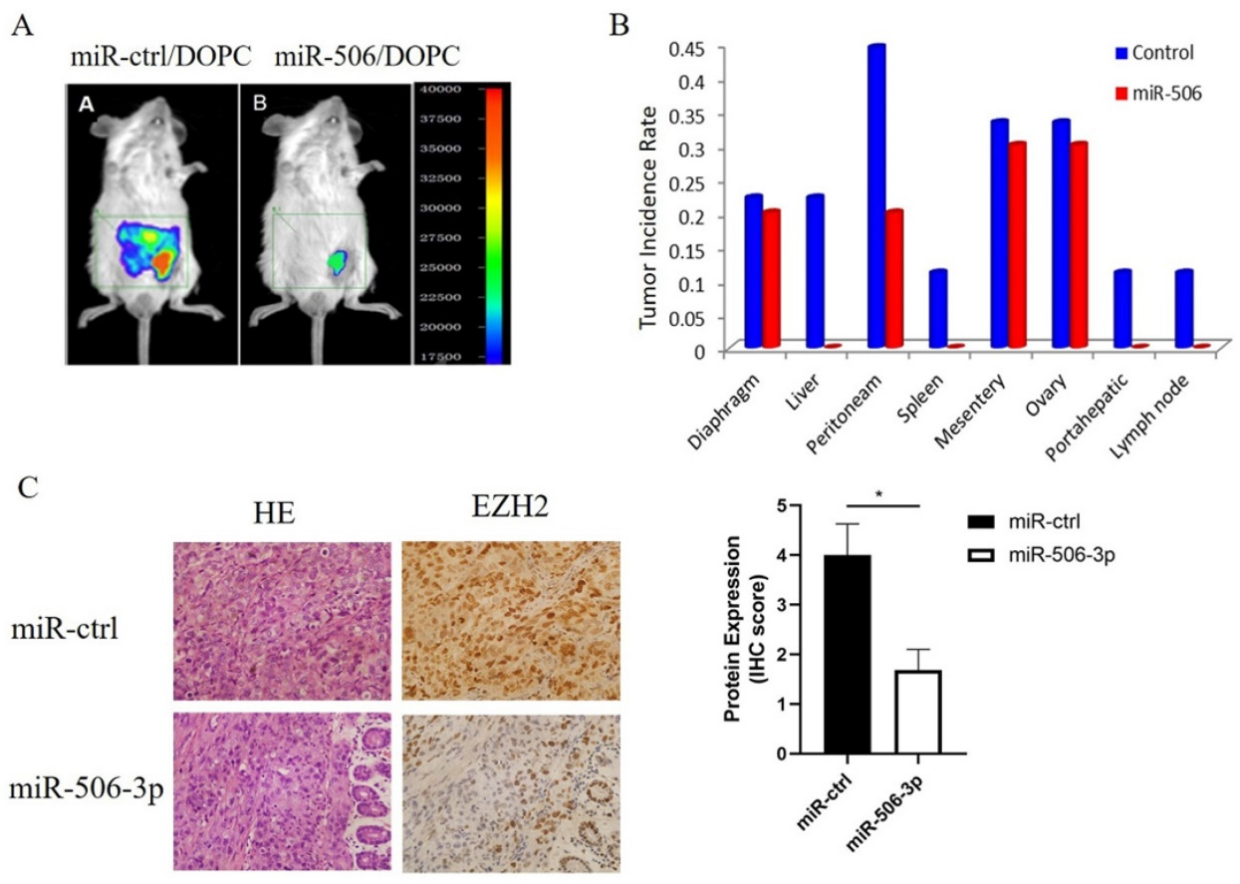
** MiR-506-3p suppresses OvCa tumor metastasis of an orthotopic mouse model of ovarian cancer.** HeyA8 cells were injected intraperitoneally into a nu/nu mouse, and an aggressive HeyA8 clone generated from ascites was developed for subsequent use (HeyA8ip1). The mice were randomly divided into control miRNA+DOPC and miR-506-3p+DOPC groups after intraperitoneal injection of HeyA8ip1 cells. (A) In vivo imaging confirmed that miR-506/DOPC nanoliposomes can inhibit *OvCa* tumor metastasis. (B) Comparison of the incidence of metastases in various organs. (C) Evaluation of miR-506-3p expression in HeyA8-ip1 tumors from control and miR-506-3p treated mice by miRNA in situ hybridization. EZH2 IHC staining was performed on HeyA8-ip1 tumor samples from control and miR-506-3p treated mice. The expression of EZH2 was calculated as the IHC staining score.

Next, we tested whether miR-506-3p could play an important role in tumor metastasis of orthotopic OvCa mouse models. In our previous study, HeyA8 cells were injected intraperitoneally into a nu/nu mouse. And then an aggressive HeyA8 clone generated from ascites was developed for subsequent use (HeyA8ip1). MiRNA was incorporated into neutrally charged DOPC nanoliposomes^30^. After intraperitoneal injection of HeyA8ip1 cells, the mice were randomly divided into two groups: 1) control miRNA+DOPC, 2) miR-506-3p+DOPC. Previous studies have reported that miR-506-3p suppressed tumor growth in orthotopic OvCa mouse models. Then, we sampled mouse abdominal organs such as peritoneam, diaphragm, liver, spleen, and lymph nodes to explore the role of miR-506-3p in ovarian cancer metastasis. As compared with the control miRNA group, the incidence of metastasis in various organs in the miR-506 group was statistically significantly decreased (Figure 4A, B). Moreover, reduced EZH2 protein was confirmed in miR-506-3p-overexpressed tumors by IHC staining (Figure 4C). In brief, the above results suggested that stable miR-506-3p overexpression inhibited the metastasis of OvCa cells in the orthotopic OvCa mouse model.

### Clinical cases confirm a negative correlation between miR-506-3p and EZH2 in OvCa

**Figure 5:**
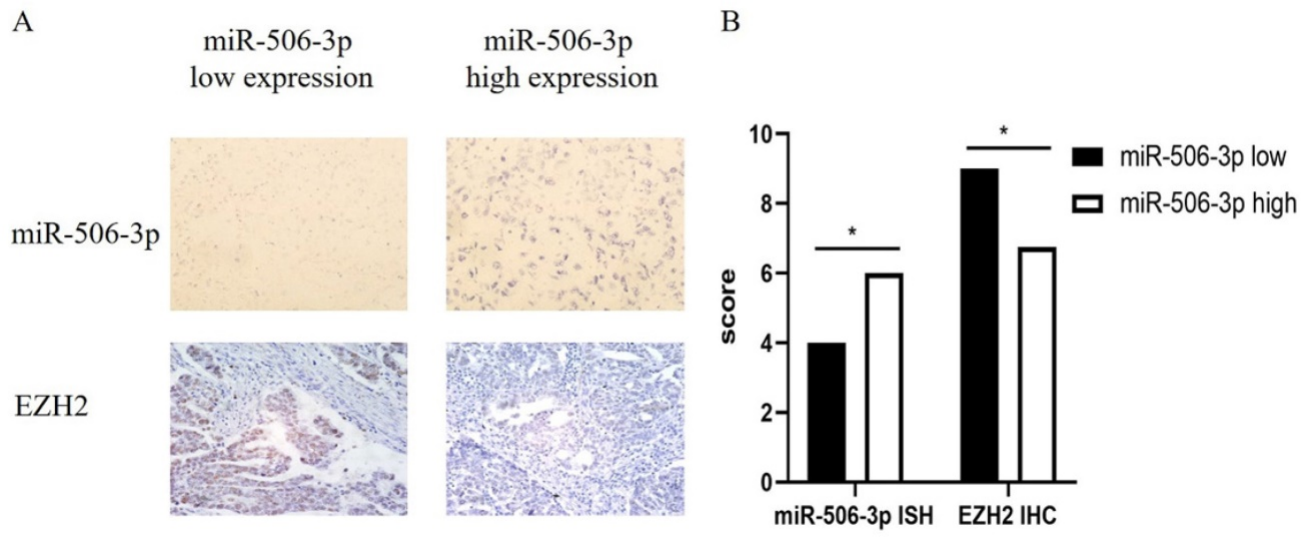
** Clinical cases confirm a negative correlation between miR-506-3p and EZH2 in OvCa.** (A) The expression of miR-506-3p in 100 OvCa patients was evaluated by miRNA in situ hybridization. OvCa samples from 100 patients were immunohistochemically stained for EZH2. (B) The expression of EZH2 was calculated as the IHC staining score.

We collected the pathological data of 100 HGS-OvCa patients and made paraffin sections. In order to study the relationship between miR-506-3p and EZH2 expression, we used IHC staining to examine the EZH2 expression and performed miRNA in situ hybridization to detect the expression of miR-506-3p. The results demonstrate that miR-506-3p is negatively correlated with the EZH2 expression in OvCa patients (Figure 5).

## Discussion

Recently, increasing evidence indicates that miRNAs play a vital role in the pathogenesis of cancer and are related to many biological processes of various tumor types, including metastasis. Invasive tumor cell metastasis is an important step in the process of cancer progression, which helps to form secondary tumors at a distance. Data has demonstrated that some miRNAs, such as miR-126, miR-145 and miR-335, are metastasis inhibitors 31. These studies have brought new insights into the use of miRNA-targeted strategies for high-metastatic tumors including OvCa.

Disorders of miR-506-3p have been identified in lung cancer, clear cell renal cell carcinoma, bladder cancer, breast cancer and cervical cancer, suggesting that miR-506-3p is obviously involved in the occurrence and progression of tumors 18-22. A previous study by Yang showed that miR-506 was reduced in clear cell renal cell carcinoma and decreased cell growth and metastasis by targeting FLOT1 20. Hou believed that miR-506 was a candidate tumor suppressor for human bladder cancer and also found that miR-506 suppressed cell proliferation, invasion, migration and EMT through targeting RWDD4 19. Further studies showed that the expression of miR-506 was reduced in 80% of cervical cancer samples and could inhibit cervical cancer growth 21. Arora reported that miR-506 was decreased in breast cancer samples and miR-506 overexpression inhibited TGF β-induced EMT, migration, and invasion in MDA-MB-231 cells 22. These results have indicated that miR-506 plays a key role in tumor progression. Our previous research initially explored the role of miR-506 in OvCa. MiR-506-3p can improve the response to chemotherapy and PARPi by regulating RAD51 homologous recombination and EZH2/β-catenin pathway and may serve as a potential therapeutic target for serous OvCa 29, 30. Besides the effects on chemotherapy sensitivity, we wonder whether miR-506-3p affects other biological process of OvCa. A study showed that miR-506-3p suppressed cell proliferation and induces senescence in OvCa by regulating the CDK4/6-FOXM1 axis 23. In addition, metastasis is a major concern in the clinical treatment of OvCa. An issue worthy of consideration is whether miR-506-3p could also affect OvCa metastasis. In our study, Transwell and wound healing assays were performed to evaluate the role of miR-506-3p in OvCa cell migration and invasion. As expected, miR-506-3p significantly reduced the migratory and invasive capacities of OvCa cells. Moreover, miR-506-3p suppressed OvCa tumor metastasis in vitro nude mice. The results might prove that miR-506-3p is a tumor suppressor in OvCa metastasis, and provide more cogent evidence supporting miR-506-3p as a promising therapeutic target in OvCa. However, the underlying mechanism needs to be further explored.

MiR-506-3p has multiple targets, through which it can regulate the biological behaviors of many cancer cells. For instance, miR-506-3p inhibits proliferation and induces senescence via decreasing the CDK4/6-FOXM1 axis directly, and increases the response to chemotherapy and PARPi by regulating RAD51-homologous recombination and EZH2/β-catenin pathway in serous OvCa 29, 30. Furthermore, Zhang proved that miR-506 was decreased in colon cancer cells and tissues, and exogenous miR-506 suppressed tumor proliferation and metastasis of colon cancer by directly targeting EZH2 27. To study the inhibitory mechanism of miR-506-3p in OvCa metastasis, we used several prediction programs to seek potential targets of miR-506-3p. Markedly, TargetScan 6.0 and luciferase reporter assay confirmed that vimentin and EZH2 are the targets of miR-506-3p in our previous studies 24, 29. We have proven that miR-506-3p could regulate E-cadherin and vimentin/N-cadherin to inhibit EMT and metastasis of OvCa, nevertheless, we cannot completely rule out the possibility that EZH2 mediate OvCa metastasis.

EZH2 is a histone methyltransferase which mediates gene silencing by catalyzing the trimethylation of histone H3 of Lys 27 (H3K27Me3) through its C-terminus SET (Su(var)3-9, Enhancer-of-zeste and Trithorax) domain 32. Through mediating the expression of critical genes, EZH2 actively participates in fundamental cellular processes such as cell proliferation, cell differentiation, cell cycle progression, and apoptosis. Mounting evidences show that EZH2 is upregulated in multiple malignancies, such as osteosarcoma, breast cancer, melanoma, prostate cancer, and OvCa, and its overexpression is related to the metastatic ability of some aggressive tumors 33-38. As for the role of EZH2 in OvCa, studies have reported that EZH2 upregulation could promote invasion and metastasis by regulating TGF-β-induced EMT, TIMP2 expression and tumor angiogenesis in OvCa 25. Consistent with those studies above, we silenced EZH2 by specific RNAi and found that EZH2 knockdown inhibited the invasion of OvCa cells.

It has been reported that miR-506/EZH2 is deregulated in colon cancer and correlated with proliferation and metastasis 27. In our experiments, we evaluated whether miR-506-3p can suppress OvCa metastasis by inhibiting the expression of EZH2 in OvCa cells. Further rescue experiments indicated that EZH2 knockdown mimicked the tumor inhibitory effects of miR-506-3p in cell models, while EZH2 overexpression obviously rescued the inhibitory effects of miR-506-3p. The orthotopic OvCa mouse models and clinical cases also confirmed the negative correlation between miR-506-3p and EZH2 in OvCa. To conclude, these results suggest that miR-506-3p modulates OvCa cell migration and invasion by targeting EZH2 directly. According to large-scale data analyses, cancer metastasis is determined to be a complex process driven by more than 100 genes 39, 40. The gain of invasiveness in OvCa cells is accompanied by the loss of epithelial characteristics and the acquisition of mesenchymal phenotype, a process known as EMT 41. Previous studies have shown that by targeting SNAI2 in OvCa, miR-506-3p increased E-cadherin expression and prevented TGF β-induced EMT, which was also regulated by EZH2 24, 25, 28. Whether and how EZH2 participates in miR-506-3p-mediated EMT regulation remains to be further studied. However, the characterization of EZH2 function in this study has inproved our understanding of OvCa metastasis to a certain extent.

In conclusion, miR-506-3p overexpression suppresses cell migration and invasion of OvCa by reducing EZH2 expression, suggesting the underlying mechanism for miR-506-3p to mediate cancer metastasis. Our results further indicate the important role of miR-506-3p in OvCa metastasis and provide more reliable evidence for the application of miR-506-3p in OvCa therapy.

## Supplementary Material

Supplementary figure and table.
