# Supplementary material for: MicroRNA-506-3p inhibits ovarian cancer metastasis by down-regulating the expression of EZH2
Source: J Cancer. 2022 Jan 4;13(3):943–50. doi: 10.7150/jca.66959 (PMC8824902; doi:10.7150/jca.66959)

Supplementary material

Table S1 Clinicopathologic information of the HGS-OvCa patients

Number	100
Age	58
Stage	
I	9
II	17
III	65
IV	9
Tumor grade	
G2	48
G3	52
Surgical	
optimal	55
suboptimal	45
Vital status	
living	52
deceased	48
Follow up	
median (range)	25 (1-79)

Figure S1. Relative levels of miR-506 in several ovarian cancer cell lines. The level of miR-506 in HeyA8 was taken as 1.

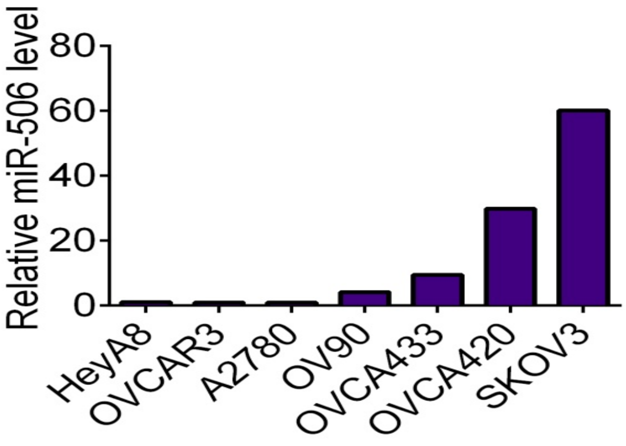

Supplement: Supplementary file 1 — Supplementary figure and table. [file jcav13p0943s1.pdf]
